# Using multi theory model (MTM) of health behavior change to explain intention for initiation and sustenance of the consumption of fruits and vegetables among African American men from barbershops in Mississippi

**DOI:** 10.34172/hpp.2020.33

**Published:** 2020-07-12

**Authors:** Jaelrbreiret L. Williams, Manoj Sharma, Vincent L. Mendy, Sophia Leggett, Luma Akil, Samuel Perkins

**Affiliations:** ^1^Department of Behavioral & Environmental Health, School of Public Health, Jackson State University, Jackson MS, USA; ^2^Department of Epidemiology and Biostatistics, School of Public Health, Jackson State University, Jackson MS, USA; ^3^Department of Business Administration, College of Business, Jackson State University, Jackson MS, USA

**Keywords:** Fruit, Vegetables, African Americans, Mississippi, Behavior

## Abstract

**Background:** African American men have poorer health outcomes compared to their white counterparts despite medical advancements and early detection of diseases. The purpose of this study was to determine to what extent the constructs of the multi theory model (MTM) explain the intention for initiation and sustenance of the consumption of fruits and vegetables among African American adult men in Mississippi.

**Methods:** Using a cross-sectional design a valid and reliable paper survey was administered during November and December of 2019. The target population for the study consisted of African American adult men (18 or older) that had not consumed recommended levels of fruits and vegetables within 24 hours of taking the questionnaire. A convenience quota sample of African American men from select barbershops in Jackson, Mississippi, were asked to complete the 40-item questionnaire on preventive health screening behavior (n=134).

**Results:** The mean total number of fruits and vegetables consumed by participants within 24hours of the taking the survey was 1.63 (SD =1.47). The mean intention to initiate consuming 5or more cups of fruits and vegetables per day score was 2.13 (SD=1.17) as measured on a 5-point scale (0-4). Behavioral confidence (β = 0.495, P<0.0001), and changes in physical environment(β = 0.230, P<0.0001) accounted for 40.8% of the variance in predicting the intention to initiate behavioral change regarding the daily consumption of fruits and vegetables. Practice for change (β = 0.462, P<0.001) and emotional transformation (β = 0.215, P<0.0001) accounted for 37.5% of the variance in the intention to sustain fruits and vegetables consumption behavior.

**Conclusion:** Based on data found in the study, MTM appears to predict the intention to initiate and sustain fruit and vegetable intake of African American men. Further research studies of suitable interventions to target African American men are needed.

## Introduction

Fruits and vegetables are a source of vitamins, minerals, antioxidants, and phytochemicals, which combat non-communicable diseases.^1^ According to the World Health Organization )WHO(, in 2019, approximately 1.7 million (2.8%) of deaths worldwide were attributable to low fruits and vegetables consumption.^2^ One of the top ten risk factors associated with global mortality is low levels of fruits and vegetables intake.^2^

In the United States, only one in 10 adults consume the recommended daily servings of one and a half to two cups of fruits and two to three cups of vegetables.^3^ Increased risk of death, vascular disease, and cancer are associated with low consumption of fruits and vegetables.^4^ Healthy People 2020 has outlined Objectives: “Nutrition and Weight Status (NWS)-14 Increase the contribution of fruits to the diet of the population age two years and older. NWS-15: Increase the participation of total vegetables to the diets of the population aged two years and older”. ^5^ Even when made aware of the health benefits of consuming fruits and vegetables African American men are less likely to eat fruits and vegetables than their white counterparts.^6^ In 2018, only 12.2% of US adults met the daily fruit intake recommended, and only 7.3% of US adults met the daily vegetable intake recommended.^3^ Only 15.8% of African Americans, non-Hispanics, consumed five or more fruits and vegetables per day.^7^ Additionally, in Mississippi, in 2017, 52% of men consumed fruits less than one time per day, while 23.6% consumed vegetables less than one time per day.^8^ Lastly, in the same year, 46.9% of African Americans in Mississippi consumed fruits less than one time per day, and 29.2% of African Americans in Mississippi consumed vegetables less than one time per day.^8^

A major public health initiative focuses on the health and wellbeing of males, but few interventions target or include the health of African American men.^9^ A few studies have been conducted to promote dietary changes among African American men.^10-13^ To our knowledge, only one study was specifically designed to promote the fruits and vegetable consumption of African American men and this study utilized the Health Belief Model and the Transtheoretical Model. ^12^ The study included African American (mostly immigrant) men from New York City in a randomized control trial designed to promote education, awareness, and adoption of fruits and vegetables among them.^12^

The multi-theory model (MTM) for health behavior change is a fourth-generation theory. MTM is parsimonious, flexible, encourages both one-time change and long-term behavior change, and it is exclusive to health education.^14^ MTM initiation constructs include *participatory dialogue* (two-way dialogue of advantages and disadvantages of behavioral change), *behavioral confidence* (confidence that the behavioral change can be continued in the future), and *changes in the physical environment* (change physical environment to allow resource readiness). MTM sustenance constructs include *emotional transformation* (ability to redirect emotions or feelings to accomplish behavioral change), *practice for change* (constantly thinking of behavior change to overcome barriers), and *changes in the social environment* (establishment of social support).^14^ MTM does not have a moderator variable; therefore, it is easily adaptable for various health behaviors. Additionally, MTM allows health practitioners the flexibility to adapt interventions to individual client needs.^15^ This study provides evidence to help guide future intervention development to promote fruit and vegetable intake of African American men helping to close the gap of knowledge. The purpose of this study was to determine to what extent the constructs of the MTM explain the intention for initiation and sustenance of the consumption of fruits and vegetables among African American adult men in Mississippi.

## Materials and Methods

### Study design, sampling, and procedure

A cross-sectional design was used to obtain data from a convenience quota sample of African American adult men from urban barbershops in Jackson, Mississippi, USA. After permission was granted from five barbershops in Jackson, Mississippi, barbershop patrons were invited to voluntarily complete a questionnaire. The target population for this study consisted of African American adult men (18 or older) who had not consumed fruits and vegetables within 24 hours of taking the survey. Data were collected between November and December 2019. The minimum sample size of 114 was calculated using G*Power with an alpha of ≤0.05, power as 0.80, three predictors (three constructs in each model) and estimated effect size of 0.10 (medium).^16^ The first author of this study visited each barbershop. The researcher approached barbershop patrons as they visited and explained the purpose of the research, if they agreed they were recruited in this study. Participants received a $5.00 voucher for a barber service if the took the survey.

### Instrumentation 

The instrument utilized for this study was a validated 40-item, face, content, and construct valid questionnaire using the MTM as the framework adapted to the African American men by the researchers. The first three questions were screening questions. Participants who disclosed eating five cups or more fruits and vegetables per day and did not identify as an African American or Black man were not included in the study. The second section of the questionnaire included eight questions about socio-demographic information. The third section of the survey contained 29 questions that measured the MTM constructs for the two models: initiation and sustenance models.

### Initiation model 

*Participatory dialogue* advantages were measured using five items that asked participants “If you eat five cups of fruits and vegetables every day you will be healthy, have variety in meals, manage your weight, have more energy, have tasty food”. All five items were deemed valid with Cronbach’s alpha of (0.77), therefore no items were removed. Advantages for fruits and vegetable intake were measured by five items with each item scored on a 5-point Likert scale (0 = never to 4 = always). The sum of the individual responses for the advantage’s items had a possible score ranging from 0–20 units. Positive scores for participatory dialogue advantages resulted in a mean score of 10 or more. *Participatory dialogue* disadvantages were measured using five items that asked participants “If you eat five cups of fruits and vegetables every day you will not have enough proteins in your diet, be hungry most of the time, have less energy, have more food related expenses, enjoy meals less”. Disadvantages for fruits and vegetables intake were measured by five items and each item was scored on a 5-point Likert scale (0= never to 4 = always). All five items were deemed valid with Cronbach’s alpha of (0.80), therefore no items were removed. The sum of the individual responses for the disadvantage’s items had a possible score ranging from 0–20 units. Positive scores for participatory dialogue disadvantages resulted in a mean score of 10 or more. The total participatory dialogue score was derived by subtracting disadvantages score from the advantages score so it had a range of -20 to +20 units. Positive scores for total participatory dialogue resulted in a mean score of 10 or more.

*Behavioral confidence* was measured using five items. Participants were asked about their level of certainty to consume five cups of fruits and vegetables daily while maintaining a budget, enjoying their meal, and not becoming fed up and without being hungry. All five items were deemed valid with Cronbach’s alpha of (0.86), therefore no items were removed. Each item was scored on a 5-point Likert scale (0 = not at all sure to 4 = completely sure). The sum of the individual responses had a possible score ranging from 0–20 units. Positive scores for behavioral confidence resulted in a mean score of 10 or more.

*Changes in the physical environment* was measured using three items that asked participants about their level of certainty to be able to consume five cups of fruits and vegetables at restaurants and being able to afford them. All three items were deemed valid with Cronbach’s alpha of (0.72), therefore no items were removed. Each item was scored on a 5-point Likert scale (0 = not at all sure to 4 = completely sure). The sum of the individual responses had a possible score ranging from 0–12 units. Positive scores for changes in the physical environment resulted in a mean score of 6 or more.

The *Intention to initiate fruits and vegetable intake* each day was measured by asking participants: “How likely is it that you will eat five cups of fruits and vegetables in the upcoming week?” The responses were scored on a 5-point Likert scale (not at all likely = 0 to completely likely = 4; a possible range of 0-4 units). Positive scores for the intention to initiate fruits and vegetable intake resulted in a mean score of two (2) or more.

The total number of items for the entire initiation scale was 18 and all items were deemed valid with a Cronbach’s alpha of (0.79), therefore no items were removed.

*Sustenance model*

*Emotional transformation* was measured using three items that asked participants about their level of certainty of directing feelings/emotions to the goal of eating five cups of fruits and vegetables every day, motivating themselves to eat five cups of fruits and vegetables each day, and overcoming self-doubt in accomplishing the goal of eating fruits and vegetables every day. All three items were deemed valid with Cronbach’s alpha of (0.88), therefore no items were removed. The responses were scored on a 5-point Likert scale (not all sure = 0 to completely sure = 4). The sum of the individual responses had a possible score ranging from 0–12 units. Positive scores for emotional transformation resulted in a mean score of six or more.

*Practice for change* was measured using three items that asked the participants about their level of surety of the following: (1) keeping a self-diary to monitor eating five cups of fruits and vegetables every day; (2) ability to eat five cups of fruits and vegetables every day if they encountered barriers; and (3) the ability to change plans for eating five cups of fruits and vegetables every day if they faced difficulties. All three items were deemed valid with Cronbach’s alpha of (0.85), therefore no items were removed. Each item was measured on a 5-point Likert scale (0 = not at all sure to 4 = completely sure). The sum of the individual responses had a possible score ranging from 0–12 units. Positive scores for practice for change resulted in a mean score of six or more.

*Changes in the social environment* was measured using three items that asked participants about their level of the surety of asking for help from a family member, friend, and a health professional to support the consumption of five cups of fruits and vegetables. All three items were deemed valid with Cronbach’s alpha of (0.77), therefore no items were removed. Each item was scored on a 5-point Likert scale (0 = not at all sure to 4 = completely sure). The sum of the individual responses had a possible score ranging from 0–12 units. Positive scores for changes in the social environment resulted in a mean score of six or more.

The *Intention to sustain fruits and vegetables intake* each day was measured by asking participants: “How likely is it that you will eat five cups of fruits and vegetables from now on? The responses were scored using a 5-point Likert scale (not at all likely = 0 to completely likely = 4 with a possible range of 0-4 units). Positive scores for the intention to sustain fruits and vegetable intake resulted in a mean score of two or more.

The total number of items for the entire sustenance scale was nine and all items were deemed valid with a Cronbach’s alpha of (0.90), therefore no items were removed.

### Statistical analysis 

The IBM SPSS Statistics, version 26 (Chicago, IL, USA) was used to analyze the data for the study. Frequencies and percentages were reported for categorical variables, and means (standard deviations [SD]) for continuous variables. Stepwise multiple regression for model building was conducted to explain behavior change while controlling for demographic variables. Two separate regression models were built for initiation model and sustenance model. The primary predictive initiation variables were participatory dialogue, behavioral confidence, and changes in the physical environment. The primary predictive sustenance variables were emotional transformation, practice for change, and changes in social environment. The *a priori* criterion of the predictor to enter into the model was *P* ≤ 0.05, and for removing, the predictor was *P* > 0.10, as is the norm in SPSS default.

## Results

### Data screening and demographics 

Total respondents of the survey were 143 of which 134 met the inclusion criteria, namely being 18 years or older, being African American men, and having not consumed 5 or more cups of fruits and vegetables within the past 24 hours. The mean age of the participants was 33.83 years with a standard deviation of 10.80. In terms of marital status (59.7%) participants reported never being married, while (28.4%) reported being currently married. More than a third (39.5%) reported living arrangements to be rented for cash and (37.3%) lived in a home that was owned by them or someone living in the household, (39.6%) reported a household income of over $50 000 while (17.9%) reported and household income of less than $10 000. Six out of 10 (60.5%) of participants reported that they were employed for wages while only 4.5% reported that they were unemployed. More than half (54.5%) reported having earned a high school diploma or general education diploma (GED); 7.5% reported to not have finished high school. Socio-demographic characteristics of the participants are presented in Table 1.

### Multi theory constructs

Table 2 depicts the descriptive statistics of study variables. For the construct of advantages, the mean score of 14.66 (standard deviation [SD]: 3.52 indicated that the participant’s attitude toward the daily consumption of five cups of fruits and vegetables was rather positive and could be beneficial for their health. The mean score of 8.18 (SD: 4.44) for construct of disadvantages showed that participant’s attitudes toward the daily consumption of five cups of fruits and vegetables leaned toward disadvantages. The mean score of the total *participatory dialogue* (advantages – disadvantages) was 6.47 (SD: 5.94) demonstrated that participants believed that the advantages outweighed the disadvantages of the consumption of five cups of fruits and vegetables. The mean score for *behavioral confidence* was 9.73 (SD: 4.77) indicated that the participants were moderately sure of their decision to initiate a change in behavior. The mean score of *changes in the physical environment* of 7.06 (SD: 3.04) indicated that participants were moderately sure of being able to consume five cups of fruits and vegetables available in their physical environment. The mean score of the *intention for initiation of behavioral change* 2.13 (SD: 1.17, median 2, range 0-4) indicated that participants were moderately likely to consume five cups of fruits and vegetables in the coming weeks.

The mean intention of sustenance of consuming five or more fruits and vegetables per day score was 1.92 (SD: 1.22). The mean score of the *emotional transformation* was 7.32 (SD: 3.26) indicated participants were moderately sure that they could encourage themselves and redirect their emotions/feelings toward a lifestyle modification to sustain the daily consumption of five cups of fruits and vegetables. The mean score of *practice for change* was 5.33 (SD: 3.28) demonstrated that the participants were slightly sure that they could engage in activities that encourage the maintenance of behavioral change in the daily consumption of five cups of fruits and vegetables. The mean score for *changes in the social environment* 6.37 (SD:3.11) indicated participants were moderately sure that they would benefit from the support of family members, friends, and health professionals in the effort to consume five cups of fruits and vegetables daily. The mean score of the *intention for sustenance of behavioral change* 1.92 (SD: 1.22, median 2, range 0-4) indicated that the participants were moderately likely to sustain the daily consumption of five cups of fruits and vegetables every week from now on.

### Initiation model 

*Behavioral confidence* (β = 0.495, *P* < 0.0001) and *changes in the physical environment* (β = 0.230, *P* < 0.0001) were significant predictors for the intention to initiate behavior change regarding fruits and vegetables consumption of African American adult men, F (2, 131) = 46.779, *P* < 0.0001; R^2^ = 0.417; adjusted R^2^ = 0.408. The results of the stepwise regression analysis to predict the intention for initiation of fruits and vegetable consumption are presented in Table 3.

### Sustenance model 

*Practice for change* (β = 0.462, *P* < 0.0001), and *emotional transformation* (β = 0.215, *P* = 0.016), were significant predictors for the intention to sustain behavior change regarding fruits and vegetables consumption of African American men, F (2, 131) = 40.984, *P* < 0.0001; adjusted R^2^ =0.375. The results of the stepwise regression analysis to predict the sustenance of fruits and vegetable consumption are presented in Table 4.

## Discussion

The purpose of this study was to examine the utility of MTM of health behavior change in predicting the initiation and sustenance of daily consumption of five cups of fruits and vegetables among African American men from barbershops in Mississippi. The results of the study revealed most MTM constructs were significant in explaining intention for fruits and vegetable consumption in African American men.

*Behavioral confidence* and *changes in the physical environment* accounted for 40.8% of the variance in predicting the intention to initiate behavioral change regarding the daily consumption of fruits and vegetables. These findings are consistent with previous studies^12,18^ that indicate that positive attitudes, control over behavior, and environmental factors are significant predictors of fruits and vegetable consumption. A similar study with African American women using the MTM showed results that all initiation constructs were significant predictors of fruit and vegetable intake.^19^ The construct of *participatory dialogue* was not a significant predictor of the initiation of fruits and vegetable intake of African American men. This may have been because the men in the sample already indicated that the advantages far outweighed the disadvantages. However, there is an opportunity to improve this further by conducting MTM interventions that target African American adult men. *Practice for change* and *emotional transformation* accounted for 37.5% of the variance in the intention to sustain fruit and vegetable consumption behavior and this finding is consistent with previous studies.^17^ A similar study with African American women using the MTM showed results that all sustenance constructs were significant predictors of fruit and vegetable intake.^19^ We did not find *changes in the social environment* as a significant predictor of the intention for sustenance of fruit and vegetable intake of African American men in this sample. This may be due to African American adult men being individuals who are determined to make their own decisions despite being advised otherwise. Additionally, African American adult men are more likely to seek a new social group with a common goal in order to reach new goals. In addition, for dietary behaviors perhaps men in particular do not like to value other people’s opinions. MTM can be used to strengthen these constructs in educational interventions by activities that involve journaling, use of apps to monitor one’s behavior, directing emotions toward goal setting and other such means. Interventions that modify these constructs are vital for helping African American men change their fruits and vegetable consumption behavior. There is a definitive need to improve these constructs through interventions. Both educational and policy interventions in this regard can be helpful. Therefore, our findings in this study lend support that some constructs of MTM may be useful to develop interventions to improve fruits and vegetables intake among African American adult men.

### Limitations/Recommendations for future research

There were some limitations to our study. First, this study utilized a cross-sectional design which cannot establish a causal relationship between the independent and dependent variables as the data was are measured at one time (snapshot). Secondly, this study utilized self-reported data, which may have been subject to recall bias, dishonesty, acquiescence bias and other such shortcomings. In addition, this study utilized the intention of behavior change for initiation and sustenance as substitution measures for actual change. The study also did not establish test-retest reliability of the survey. Lastly, random sampling was not utilized; therefore, the results of this survey are limited by the choice of the sample, which limit generalizability.

Future research should be longitudinal and experimental to establish causality between MTM constructs and fruits and vegetables consumption. For future cross-sectional survey research, participants should be randomly selected to improve generalizability. Additionally, researchers should not only utilize self-reported data but measure participants objectively and through observation of fruits and vegetable consumption behavior. Experimental designs should be used to develop and test MTM-based interventions for modifying the constructs identified in this study for promoting fruits and vegetable consumption among African American men. This study paves way for undertaking a series of pilot interventional studies followed by efficacy trials and then possibly effectiveness studies to promote fruits and vegetable consumption behavior among African American men.

### Implications for practice 

Results of this study support MTM being operational in designing and evaluating interventions that promote fruits and vegetable consumption among African American adult men. *Participatory dialogue* can be influenced by engaging in two-way discussions about the advantages of consuming five cups of fruits and vegetables. We can utilize barbershops as a great place to engage in the two-way conversations facilitated by certified health education specialists or other health professionals. We can influence *behavioral confidence* by having food demonstrations outside of barbershops and providing tips on quick meals and choosing food options that would help men consume fruits and vegetables. Influencing the construct of *changes in the physical environment* would involve making more fruits and vegetables available (i.e., healthy snack options in the barbershops and in the community through resource mobilization and policy efforts). To help make long-term change of African American adult men consuming five or more fruits and vegetables, participants would need to transform emotions (*emotional transformation* construct) toward eating five cups of fruits and vegetables. We can accomplish this by discussions regarding goal setting with health professionals about food substitution for fruits and vegetables as well as setting up barbershop talks with community health educators. The *practice for change* construct can be improved by teaching the African American adult men to monitor their behavior by keeping an electronic journal (e.g. “take a pic before your pick it”) to help them adhere to their goals. We can influence *changes in social environment* by getting support of family, friends or health professionals and can occur naturally or artificially.

## Conclusion

This cross-sectional study provided evidence in favor of the multi-theory model (MTM) of health behavior change in helping promote change with regard to fruits and vegetable consumption behavior among African American men. We can reify this theory for developing interventions to facilitate fruits and vegetable consumption in African American men. A venue for such interventions can be barbershops.

## Ethical approval

This study was approved by Jackson State University Institutional Review Board (IRB), Protocol #0149-19. All procedures performed in this study were in accordance with ethical standards of the institutional and or national research committee.

## Competing interests

None to declare.

## Funding

The authors received no financial support for research, authorship, and/or publication of this article.

## Authors’ contributions

Manuscript conceptualization: JLW and MS; Manuscript writing: JLW, MS, VLM, SL, LA, SP; Literature review: JLW, MS, VLM, SL, LA; Instrument development; MS; Data Collection: JLW; Data analysis: JLW and MS; Data interpretation: JLW and MS.

**Table 1:** Socio-demographic Characteristics of the Participants (n = 134)

**Variable**	
Age (y) [Mean (SD)]	33.83 (10.80)
Marital status, No. (%)	
Now Married	38 (28.4)
Widower	1 (0.7)
Divorced	12 (9.0)
Separated	3 (2.2)
Never married	80 (59.7)
Living arrangements, No. (%)	
Owned by you with mortgage	50 (37.3)
Owned by you or someone in the house no mortgage	19 (14.2)
Rented for cash rent	53 (39.5)
Occupied without payment of cash rent	12 (9.0)
Household Income, $, No. (%)	
Less than 10 000	24 (17.9)
10 000 to 19 999	13 (9.7)
20 000 to 29 999	17 (12.7)
30 000 to 39 999	18 (13.4)
40 000 to 49 999	9 (6.7)
Over 50 000	53 (39.6)
Employment status, No. (%)	
Student	15 (11.2)
Employed for wages	81 (60.5)
Self-employed	24 (17.9)
Unemployed	6 (4.5)
Retired	2 (1.5)
Student and employed for wages	5 (3.7)
Employed for wages and retired	1 (0.7)
Education level, No. (%)	
Did not finish high school	10 (7.5)
High school diploma/GED	73 (54.5)
Associate degree	19 (14.2)
Bachelor’s degree	19 (14.2)
Master’s degree	11 (8.2)
Professional degree	1 (0.7)
Doctorate degree	1 (0.7)
Diagnosis of disease (diabetes, heart disease, stroke, cancer, Alzheimer’s disease)	
None	124 (92.6)
Diabetes	9 (6.7)
Diabetes, heart disease, and stroke	1 (0.7)

**Table 2:** Multi theory model construct survey scores of African American men who did not consume at least five cups of fruits and vegetables daily

**Constructs**	**Possible Range**	**Observed Range**	**Mean (SD)**	**Cronbach α**
Cups of fruits^a^	0-4	0-4	0.74 (0.91)	
Cups of vegetables^a^	0-4	0-4	0.89 (1.00)	
Total cups of fruits and vegetables^a^	0-4	0-4	1.63 (1.47)	
Initiation	0 to 4	0 to 4	2.13 (1.17)	-
Participatory dialogue (PD)				
Overall PD Advantages – Disadvantages	-20 to +20	-8 .00 to 20.00	6.47 (5.94)	-
PD advantages	0 to 20	2.00 to 20.00	14.66 (3.52)	0.77
PD disadvantages	0 to 20	0.00 to 20.00	8.18 (4.44)	0.80
Behavioral confidence	0 to 20	0.00 to 20.00	9.73 (4.77)	0.86
Changes in physical environment	0 to 12	0.00 to 12.00	7.06 (3.04)	0.72
Sustenance	0 to 4	0 to 4	1.92 (1.22)	-
Emotional transformation	0 to 12	0.00 to 12.00	7.32 (3.26)	0.88
Practice for change	0 to 12	0.00 to 12.00	5.33 (3.28)	0.85
Change in social environment	0 to 12	0.00 to 12.00	6.37 (3.11)	0.77

^a^Consumed within 24 hours of completion of survey.

**Table 3:** Stepwise regression analysis to explain intention of initiation of fruits and vegetables consumption behavior change in African American men

**Constructs** ^a^	**Β**	**SE**_B_	**β**	***P*** **value**	**95% CI for B**
Model^b^					
Behavioral confidence	0.121	0.019	0.495	<0.0001	0.083, 0.159
Changes in physical environment	0.089	0.030	0.230	<0.0001	0.029, 0.148

Abbreviations: B, unstandardized coefficient; CI, confidence interval; SEB, standard of the coefficient; β, standardized coefficient. ^a^Dependent variable: How likely is it that you will eat five cups of fruits and vegetables every day in the upcoming week? ^b^ F (2, 131) = 46.779, *P* < 0.0001; R^2^= 0.417; adjusted R^2^ = 0.408.

**Table 4:** Stepwise regression analysis to explain intention to sustain fruits and vegetables consumption behavior change in African American men

**Constructs** ^a^	**Β**	**SE**_B_	**β**	***P*** **value**	**95% CI for B**
Model^b^					
Practice for change	0.172	0.033	0.462	<0.0001	0.107, 0.237
Emotional transformation	0.080	0.033	0.215	0.016	0.015, 0.146

Abbreviations: B, unstandardized coefficient; CI, confidence interval; SEB, standard of the coefficient; β, standardized coefficient. ^a^ Dependent variable: How likely is it that you will eat five cups of fruits and vegetables every day from now on? ^b^ F (2, 131) = 40.984, *P* < 0.0001; R^2^ = 0.385; adjusted R^2^ = 0.375.
